# Diagnostic Accuracy of Artificial Intelligence in Endoscopy: Umbrella Review

**DOI:** 10.2196/56361

**Published:** 2024-07-15

**Authors:** Bowen Zha, Angshu Cai, Guiqi Wang

**Affiliations:** 1 Department of Endoscopy, National Cancer Center/National Clinical Research Center for Cancer/Cancer Hospital, Chinese Academy of Medical Sciences and Peking Union Medical College, Beijing, China

**Keywords:** endoscopy, artificial intelligence, umbrella review, meta-analyses, AI, diagnostic, researchers, researcher, tools, tool, assessment

## Abstract

**Background:**

Some research has already reported the diagnostic value of artificial intelligence (AI) in different endoscopy outcomes. However, the evidence is confusing and of varying quality.

**Objective:**

This review aimed to comprehensively evaluate the credibility of the evidence of AI’s diagnostic accuracy in endoscopy.

**Methods:**

Before the study began, the protocol was registered on PROSPERO (CRD42023483073). First, 2 researchers searched PubMed, Web of Science, Embase, and Cochrane Library using comprehensive search terms. Then, researchers screened the articles and extracted information. We used A Measurement Tool to Assess Systematic Reviews 2 (AMSTAR2) to evaluate the quality of the articles. When there were multiple studies aiming at the same result, we chose the study with higher-quality evaluations for further analysis. To ensure the reliability of the conclusions, we recalculated each outcome. Finally, the Grading of Recommendations, Assessment, Development, and Evaluation (GRADE) was used to evaluate the credibility of the outcomes.

**Results:**

A total of 21 studies were included for analysis. Through AMSTAR2, it was found that 8 research methodologies were of moderate quality, while other studies were regarded as having low or critically low quality. The sensitivity and specificity of 17 different outcomes were analyzed. There were 4 studies on esophagus, 4 studies on stomach, and 4 studies on colorectal regions. Two studies were associated with capsule endoscopy, two were related to laryngoscopy, and one was related to ultrasonic endoscopy. In terms of sensitivity, gastroesophageal reflux disease had the highest accuracy rate, reaching 97%, while the invasion depth of colon neoplasia, with 71%, had the lowest accuracy rate. On the other hand, the specificity of colorectal cancer was the highest, reaching 98%, while the gastrointestinal stromal tumor, with only 80%, had the lowest specificity. The GRADE evaluation suggested that the reliability of most outcomes was low or very low.

**Conclusions:**

AI proved valuabe in endoscopic diagnoses, especially in esophageal and colorectal diseases. These findings provide a theoretical basis for developing and evaluating AI-assisted systems, which are aimed at assisting endoscopists in carrying out examinations, leading to improved patient health outcomes. However, further high-quality research is needed in the future to fully validate AI’s effectiveness.

## Introduction

Gastrointestinal diseases impose a serious burden on health care systems worldwide. The data show that gastrointestinal diseases cause millions of deaths worldwide every year [1]. Endoscopy, as an efficient and convenient method, can effectively diagnose various gastrointestinal diseases [2]. Endoscopic intervention can also effectively treat early gastrointestinal cancers [3].

In recent years, with the rise of artificial intelligence (AI), numerous studies have been conducted to explore its application in the field of endoscopy, aiming to assist medical professionals in lesion identification and endoscopy quality control [4,5].

At present, some meta-analyses have reported the diagnostic value of AI in endoscopy [6-9]. Although AI has high sensitivity and specificity in identifying lesions in some studies, due to merger heterogeneity and sample size variations, the reliability of merger analysis outcomes needs further discussion [10-12].

In this study, an umbrella review methodology was used to elucidate current research directions and identify potential future research ideas by evaluating existing meta-analyses on AI in endoscopy. The meta-analyses of current studies were screened and extracted, and the quality of outcomes was assessed.

## Methods

### Registration

The protocol was registered on PROSPERO (CRD42023483073) before the study began. PROSPERO is an open access database of systematic reviews. Registration before the start of the study effectively reduced selective reporting [13,14]. This umbrella review followed the PRISMA (Preferred Reporting Items for Systematic Reviews and Meta-Analyses) guidelines. The details can be seen in Checklist 1.

### Search Strategy

Two researchers searched PubMed, Web of Science, Embase, and Cochrane Library with a comprehensive search strategy up to November 2023. In addition, we searched “Google Scholar” to identify gray literature and searched for references of eligible articles. Two researchers independently screened the titles and abstracts and reviewed the full texts to identify eligible studies. Any discrepancies were resolved through consultation with a third researcher until a consensus was reached. The search strategy details are available in Table S1 in Multimedia Appendix 1.

### Inclusion and Exclusion Criteria

The inclusion criteria were as follows: (1) studies evaluating the diagnostic value of AI in endoscopy; (2) studies that provided at least one outcome data—sensitivity or specificity; (3) articles that had meta-analyses and were conducted by systematic methods; and (4) articles published in English.

We excluded studies that met the following criteria: (1) experiments not on humans, (2) unavailable full text, (3) duplicate studies, and (4) studies lacking critical information.

### Data Extraction

Two researchers independently extracted data. The third researcher would extract data if there were any discrepancies. The following basic information was included: the first author, year of publication, country, kind of endoscopy, detection, followed guidelines, registered number, number of included studies in the meta-analyses, outcomes, included study types in the meta-analyses, and tools for assessing the risk of the Bias. Then, we collected outcome information, including sensitivity, specificity, positive likelihood ratio, negative likelihood ratio, diagnostic odds ratio, and area under the curve. We searched for missed information in primary studies if necessary.

### Evaluation of Article Quality

Two reviewers independently evaluated the quality of the articles using A Measurement Tool to Assess Systematic Reviews 2 (AMSTAR2). AMSTAR is a tool for evaluating the systematic reviews of randomized trials [15,16]. In 2015, researchers introduced AMSTAR2, which expanded the application scope of AMSTAR to include the evaluation of systematic reviews of nonrandomized trials [17]. AMSTAR2 consists of a 16-item questionnaire prompting reviewers to respond with “yes,” “partly yes,” or “no” to each item. We viewed 2 “partly yes” answers as 1 “yes.” In total, 7 items were considered important. If all the items were in conformity or only 1 unimportant item was out of conformity, the study was evaluated as having high quality. If more than 1 unimportant item did not fit, the study was rated as having moderate quality. If 1 important item did not conform, the study was rated as having low quality; the study was regarded as having critically low quality if more than 1 important item did not conform.

### Data Analysis

We collected the outcome indicators of applying AI technology in different scenarios. This study evaluated the application of AI diagnostic techniques in different endoscopes. Considering that there are several studies analyzing the same issues, if there were multiple meta-analyses, we selected high-quality studies according to the AMSTAR2 criteria. If the quality of different studies was consistent, we chose the latest published study among them. After that, the most recent meta-analysis was collected and performed again to ensure that the most recent results were obtained. To make the results more reliable, we chose a more conservative method. Moreover, we used the random effect model to ensure the reliability of the result.

We calculated the effect quantity and 95% CI of each meta-analysis. In each meta-analysis, the *P* value of the Cochran Q test and the *I*^2^ metric were used to evaluate the heterogeneity caused by the threshold effect. The Deek test was used to test publication bias. We used forest figures to show the diagnostic value of AI in endoscopy. We also used the bar accumulation charts to show the conformity of the included articles. In this study, we used R (version 4.3.2; R Foundation for Statistical Computing) for calculation. If the *P* value was more than .05, we considered that there was no statistically significant difference.

### Grading of the Evidence

Using the Grading of Recommendations, Assessment, Development, and Evaluation (GRADE) principle, 2 reviewers evaluated the credibility of evidence independently. GRADE proposes 5 factors for downgrading certainty in the evidence (the risk of bias, inconsistency, indirectness, imprecision, and publication bias) and 2 factors for upgrading certainty in the evidence (large effect and dose-response). These factors were used to evaluate outcomes as being of high, moderate, low, or very low quality. The body of evidence for diagnostic test accuracy studies begins with high quality. There was no guidance on the up factors in the diagnostic test accuracy study; we only downgraded the evidence using the 5 downgrading factors. For the comparative study, we defined its initial reliability according to the results of AMSTAR2 and then adjusted it according to the above factors.

## Results

### Study Selection

**Figure 1.**
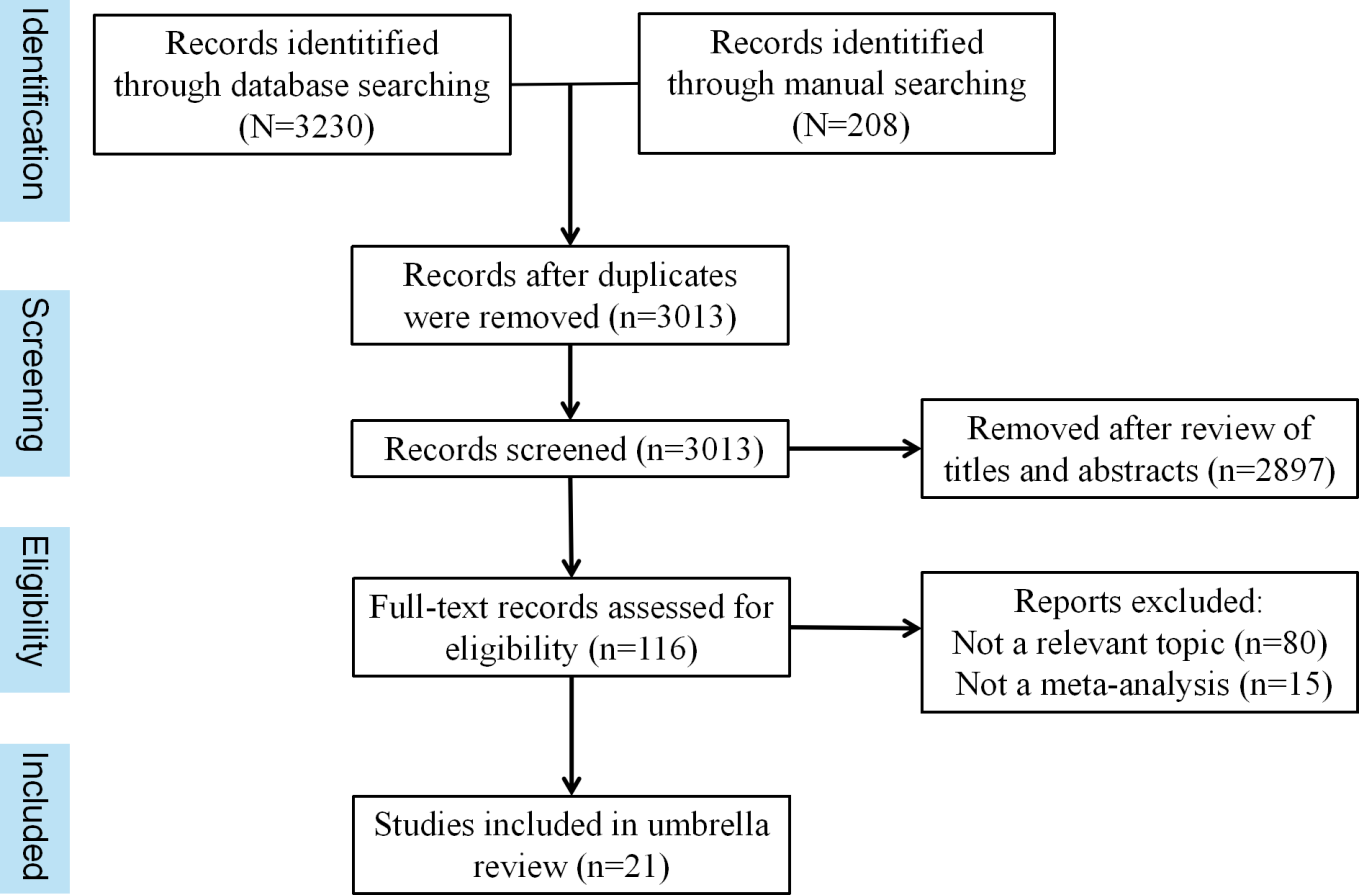
Search strategy and study screening.

We initially identified 3230 studies through the database and 208 studies through manual retrieval. After eliminating duplicates, we had 3013 studies. Then, the researchers eliminated 2897 studies that did not meet the criteria based on their titles and abstracts. After reading the full text of 116 studies, 80 irrelevant studies and 15 studies without meta-analyses were excluded, and finally, 21 studies were included for statistical analysis and evaluation. These included 10 studies pertaining to upper gastrointestinal endoscopy[9,18-26], 5 studies focusing on colonoscopy [27-31], and 4 studies on capsule endoscopy [32-35]. Additionally, there was 1 study about endoscopic ultrasound (EUS) and 1 study about laryngoscopy [36,37]. Detail can be seen in Figure 1.

### Included Study Characteristics

A total of 10 studies reported the diagnostic value of AI technoloyg in upper gastrointestinal endoscopy. These studies encompassed various original research papers, ranging from 7 to 39 studies per investigation. These studies analyzed the diagnostic value of AI in various diseases, including esophageal and gastric neoplasia, Barrett esophagus, and *Helicobacter pylori* infection. In terms of research strategies, 9 research reports followed PRISMA guidelines, and 5 studies were registered on PROSPERO. With regard to evaluating bias, 8 studies used Quality Assessment of Diagnostic Accuracy Studies 2 (QUADAS-2), 1 study used QUADAS, and 1 study was not evaluated. QUADAS evaluates the diagnostic accuracy of the research system, including patient selection, index test, reference standard, flow, and timing. In 2011, researchers developed QUADAS-2 for better evaluation [38]. All studies were included in observational studies for diagnostic evaluation.

A total of 5 research studies on the diagnostic value of AI in colonoscopy were included. Among them, 1 study focused on ulcerative colitis, one focused on colon polyps and tumors, one used Prediction Model Risk of Bias Assessment Tool (PROBAST) to evaluate bias, 3 used QUADRAS-2, and one was not evaluated.

Of the remaining 6 included studies, 4 studies reported the value of AI in capsule endoscopy for diagnosing bleeding and ulcers; 2 studies reported AI’s diagnostic value of laryngoscopes in examining normal or diseased throat structures and in EUS for diagnosing gastrointestinal stromal tumors separately; 5 studies were conducted according to the PRISMA guidelines; and 3 studies were registered in advance. All 6 studies were included in the observational study, and 5 of them used QUADRAS-2. The details can be seen in Table 1 and Table S2 in Multimedia Appendix 1.

**Table 1.** Basic information of included studies.

Study	Year	Country	Kind of endoscopy	Aim	Included studies, n	Followed guidelines
Tan et al [24]	2022	Australia	Upper endoscopy	Detection of Barrett esophagus	12	PRISMA^a^
Ma et al [22]	2022	China	Upper endoscopy	Detection of esophagus cancer	7	PRISMA
Bang et al [32]	2020	Korea	Upper endoscopy	Detection of *Helicobacter pylori* infection	8	PRISMA
Shi et al [23]	2022	China	Upper endoscopy	Detection of chronic atrophic gastritis	8	PRISMA
Guidozzi et al [20]	2023	South Africa	Upper endoscopy	Detection of Barrett esophagus and cancer	14	PRISMA
Jahagirdar et al [29]	2023	America	Colonoscopy	Detection of ulcerative colitis	12	PRISMA
Keshtkar et al [30]	2023	Iran	Colonoscopy	Detection of colorectal polyp and cancer	24	NR^b^
Bang et al [32]	2022	Korea	Wireless capsule Endoscopy	Detection of ulcers, polyps, celiac disease, bleeding, and hookworm	39	PRISMA
Soffer et al [35]	2020	Israel	Wireless capsule Endoscopy	Detection of ulcers, polyps, celiac disease, bleeding, and hookworm	19	PRISMA
Gomes et al [36]	2023	America	Endoscopic ultrasonography	Detection of gastrointestinal stromal tumor	8	PRISMA
Zurek et al [37]	2022	Poland	Laryngeal endoscopy	Detection of lesions in the larynx	11	PRISMA
Bai et al [27]	2023	China	Colonoscopy	Prediction of invasion depth of colorectal cancer or neoplasms	10	PRISMA
Qin et al [34]	2021	China	Wireless capsule endoscopy	Detection of erosion/ulcer, gastrointestinal bleeding, and polyps/cancer	16	PRISMA
Mohan et al [33]	2021	America	Wireless capsule endoscopy	Detection of gastrointestinal ulcers	9	NR
Bang et al [28]	2021	Korea	Colonoscopy	Detection of diminutive colorectal polyps	13	PRISMA
Lui et al [31]	2020	China	Colonoscopy	Detection of colorectal polyp and cancer	18	PRISMA
Lui et al [21]	2020	China	Upper endoscopy	Detection of gastric and esophageal neoplastic lesions and *Helicobacter pylori*	23	PRISMA
Visaggi et al [9]	2021	Italy	Upper endoscopy	Detection of Barrett neoplasia	19	NR
Zhang et al [26]	2021	China	Upper endoscopy	Detection of esophageal cancer and neoplasm	16	PRISMA
Xie et al [25]	2022	China	Upper endoscopy	Detection of gastric cancer and prediction invasion depth	17	PRISMA
Chen et al [19]	2022	China	Upper endoscopy	Detection of early gastric cancer	12	PRISMA

a PRISMA: Preferred Reporting Items for Systematic reviews and Meta-Analyses.

b NR: not reported.

### Methodological Quality of Included Studies

In all the included studies, methodological quality ranged from very low to moderate. Results show that the methodology was rated as moderate for 6 studies, low for 2 studies, and very critically low for the remaining 13 studies. Among the articles about upper endoscopy, it was found that 5 studies exhibited a moderate level of methodological quality. In comparison, 2 studies were deemed to have low quality, and 3 studies had were very low quality. The critical problems were the need for advanced registration and an incomplete retrieval strategy. The noncritical problem was that the original literature funding had not been reported. Besides, studies of moderate methodological quality were conducted on both the stomach and esophagus of the upper gastrointestinal tract. Three studies on colonoscopy were of moderate quality, 2 were of low quality, and the remaining 8 were of very low quality. The main problems were the meta-merging method and the evaluation of publication bias.

Regarding the application of AI in capsule endoscopy, 1 study was of moderate quality, and the other 3 had critically low quality. In addition, the research on applying EUS to identify gastrointestinal stromal tumors and laryngoscope to identify normal and pathological structures of the throat had critically low quality. The details can be seen in Table S3 and Table S4 in Multimedia Appendix 1.

### Meta-Analyses

There were 4 outcomes for the esophagus. The sensitivity was 0.89 (95% CI 0.84-0.93) for esophageal neoplasia, 0.95 (95% CI 0.91-0.98) for esophageal squamous cell carcinoma, 0.94 (95% CI 0.67-0.99) for abnormal intrapapillary loops, and 0.97 (95% CI 0.67-1.00) for gastroesophageal reflux disease. Their specificity was 0.86 (95% CI 0.83-0.93) for esophageal neoplasia, 0.92 (95% CI 0.82-0.97) for esophageal squamous cell carcinoma, 0.94 (95% CI 0.84-0.98) for abnormal intrapapillary loops, and 0.97 (95% CI 0.75-1.00) for gastroesophageal reflux disease. The sensitivity of gastric cancer and chronic atrophic gastritis was 0.89 (95% CI 0.85-0.93) and 0.94 (95% CI 0.88-0.97), respectively. At the same time, their specificity was 0.93 (95% CI 0.88-0.97) and 0.96 (95% CI 0.88-0.98), respectively. The sensitivity and specificity of judging the invasion depth of gastric cancer were 0.82 (95% CI 0.78-0.85) and 0.90 (95% CI 0.82-0.95), respectively. The sensitivity and specificity of *Helicobacter pylori* infection were 0.87 (95% CI 0.72-0.94) and 0.86 (95% CI 0.72-0.96).

In colonoscopy, the sensitivity and specificity of colon polyps were 0.93 (95% CI 0.91-0.95) and 0.87 (95% CI 0.76-0.93), respectively. The sensitivity and specificity of colon neoplasia were 0.94 (95% CI 0.85-0.98) and 0.98 (95% CI 0.94-0.99), respectively. The sensitivity and specificity of ulcerative colitis were 0.83 (95% CI 0.78-0.87) and 0.92 (95% CI 0.89-0.95), respectively. For invasion depth of colon neoplasia, the sensitivity and specificity were 0.71 (95% CI 0.58-0.81) and 0.95 (95% CI 0.91-0.97), respectively.

**Figure 2.**
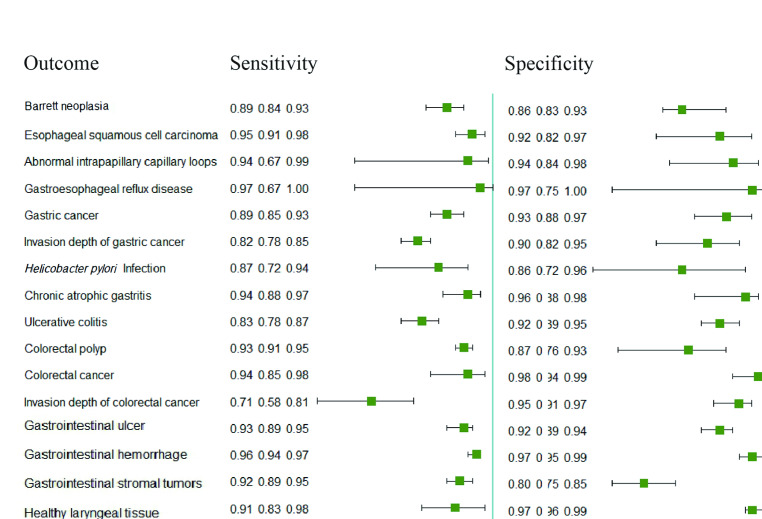
Diagnostic value of artificial intelligence in different endoscopic outcomes.

For wireless capsule endoscopy, we got 2 results. The sensitivity and specificity of the diagnosis of gastrointestinal ulcer were 0.93 (95% CI 0.89-0.95) and 0.92 (95% CI 0.89-0.95), respectively. The sensitivity and specificity of the diagnosis of gastrointestinal bleeding were 0.96 (95% CI 0.94-0.97) and 0.97 (95% CI 0.95-0.99), respectively. The sensitivity and specificity of EUS in diagnosing gastrointestinal stromal tumors were 0.92 (95 % CI 0.89-0.95) and 0.80 (95 % CI 0.75-0.85), respectively. The sensitivity of healthy and diseased tissues in AI-identified laryngoscope was 0.91 (95 % CI 0.83-0.98) and 0.91 (95 % CI 0.86-0.96), respectively, and the specificity was 0.97 (95 % CI 0.96-0.99) and 0.95 (95 % CI 0.90-0.99), respectively. The details can be seen in Figure 2 and Table 2.

**Table 2.** Outcomes of artificial intelligence in endoscopy diagnosis.

Study	Detection	Sensitivity (95% CI)	Specificity (95% CI)	PLR^a^ (95% CI)	NLR^b^ (95% CI)	DOR^c^ (95% CI)	AUC^d^ (95% CI)	Model
Tan et al [24]	Early Barrett esophagus	0.90 (0.87-0.93)	0.84 (0.80-0.88)	NR^e^	NR	0.90 (0.87-0.93)	NR	Random
Ma et al et al [22]	Early esophageal cancer	0.90 (0.82-0.94)	0.91 (0.79-0.96)	9.8 (3.8-24.8)	0.11 (0.06-0.21)	NR	0.95^f^	NR
Bang et al [18]	*Helicobacter pylori* Infection	0.87 (0.72-0.94)	0.86 (0.72-0.96)	6.2 (3.8-10.1)	0.15 (0.07-0.34)	40 (15‐112)	0.92 (0.90-0.94)	NR
Guidozzi et al[20]	Esophageal squamous cell carcinoma	0.91 (0.84-0.95)	0.80 (0.63-0.90)	NR	NR	NR	NR	Random
Guidozzi et al [20]	Esophageal adenocarcinoma	0.91 (0.87-0.94)	0.87 (0.82-0.91)	NR	NR	NR	NR	NR
Shi et al [23]	Chronic atrophic gastritis	0.94 (0.88-0.97)	0.96 (0.88-0.98)	21.58 (7.91-58.85)	0.07 (0.04-0.13)	320.19 (128.5-797.84)	0.98 (0.96-0.99)	NR
Jahagirdar et al [29]	Ulcerative colitis	0.83 (0.78- 0.87)	0.92 (0.89-0.95)	NR	NR	NR	0.92 (0.88-0.94)	NR
Keshtkar et al [30]	Colorectal polyp	0.92 (0.85-0.96)	0.94 (0.89-0.96)	14.5 (8.4-25.2)	0.09 (0.05-0.16)	162 (59.44-5)	0.97 (0.96-0.99)	NR
Keshtkar et al [30]	Colorectal cancer	0.94 (0.85-0.98)	0.98 (0.94-0.99)	41.2 (13.7-124.2)	0.06 (0.02-0.16)	677 (108-4240)	0.99 (0.98-1.00)	NR
Bang et al [32]	Gastrointestinal ulcer	0.93 (0.89-0.95)	0.92 (0.89-0.94)	NR	NR	138 (79-243)	0.97 (0.95-0.98)	NR
Bang et al [32]	Gastrointestinal hemorrhage	0.96 (0.94-0.97)	0.97 (0.95-0.99)	NR	NR	888 (343-2303)	0.99 (0.98-0.99)	NR
Soffer et al [35]	Mucosal ulcers	0.95 (0.89-0.98)	0.94 (0.90-0.96)	NR	NR	NR	NR	Random
Soffer et al [35]	Bleeding	0.98 (0.96-0.99)	0.99 (0.97-0.99)	NR	NR	NR	NR	Random
Gomes et al [36]	Gastrointestinal stromal tumors	0.92 (0.89-0.95)	0.80 (0.75-0.85)	4.26 (2.7-6.7)	0.09 (0.14-0.18)	71.74 (22.43-229.46)	0.949^f^	NR
Zurek et al [37]	Healthy laryngeal tissue	0.91 (0.83-0.98)	0.97 (0.96-0.99)	NR	NR	NR	0.945^f^	Random
Zurek et al [37]	Benign and malignant lesions	0.91 (0.86-0.96)	0.95 (0.90-0.99)	NR	NR	NR	0.924^f^	Random
Bai et al [27]	Invasion depth of early colorectal cancer	0.71 (0.58-0.81)	0.95 (0.91-0.97)	NR	NR	NR	0.93 (0.90-0.95)	NR
Qin et al [34]	Erosion or ulcers	0.96 (0.91-0.98)	0.97 (0.93-0.99)	36.8 (12.3-110.1)	0.04 (0.02-0.09)	893 (103-5834)	0.99 (0.98-1.00)	NR
Qin et al [34]	Gastrointestinal bleeding	0.97 (0.93-0.99)	1.00 (0.99-1.00)	289.4 (80.3-1043.0)	0.03 (0.01-0.08)	10,291 (1539-68,791)	1.00 (0.99-1.00)	NR
Qin et al [34]	Polyps and cancer	0.97 (0.82-0.99)	0.98 (0.92-0.99)	42.7 (11.3-161.8)	0.03 (0.01-0.21)	1291 (60-27-808)	0.99 (0.98-1.00)	NR
Mohan et al [33]	Gastrointestinal ulcers or hemorrhage	0.96 (0.94-0.97)	0.96 (0.95-0.97)	NR	NR	NR	95.4 (94.3-96.3)	NR
Bang et al [28]	Colorectal polyps	0.93 (0.91-0.95)	0.87 (0.76-0.93)	7.1 (3.8-13.3)	0.08 (0.06-0.11)	87 (38-201)	0.96 (0.93-0.97)	NR
Lui et al [31]	Colorectal polyps	0.92 (0.89-0.95)	0.90 (0.85-0.93)	NR	NR	NR	0.96 (0.95-0.98)	Random
Lui et al [21]	Neoplastic lesions in the stomach	0.92 (0.88-0.95)	0.88 (0.78-0.95)	NR	NR	NR	0.96 (0.94-0.99)	NR
Lui et al [21]	Barrett esophagus	0.88 (0.83-0.92)	0.90 (0.86-0.95)	NR	NR	NR	0.96 (0.93-0.99)	NR
Lui et al [21]	Neoplastic lesions in squamous esophagus	0.76 (0.48-0.93)	0.92 (0.67-0.99)	NR	NR	NR	0.88 (0.82-0.96)	NR
Lui et al [21]	Helicobacter pylori status	0.84 (0.71-0.93)	0.90 (0.79-0.96)	NR	NR	NR	0.92 (0.88-0.97)	NR
Visaggi et al [9]	Barrett neoplasia	0.89 (0.84-0.93)	0.86 (0.83-0.93)	6.50 (1.59-2.15)	0.13 (0.20-0.08)	50.53 (24.74-103.22)	0.90 (0.85-0.94)	Random
Visaggi et al [9]	Esophageal squamous cell carcinoma	0.95 (0.91-0.98)	0.92 (0.82-0.97)	12.65 (1.61-3.51)	0.05 (0.11-0.02)	258.36 (44.18-1510.7)	0.97 (0.92-0.98)	Random
Visaggi et al [9]	Abnormal intrapapillary capillary loops	0.94 (0.67-0.99)	0.94 (0.84-0.98)	14.75 (1.46-3.70)	0.07 (0.39-0.01)	225.83 (11.05- 4613.93)	0.98 (0.86-0.99)	Random
Visaggi et al [9]	Gastroesophageal reflux disease	0.97 (0.67-1.00)	0.97 (0.75-1.00)	38.26 (0.98-6.22)	0.03 (0.44-0.00)	1159.6 (6.12-219711.69)	0.99 (0.80-0.99)	Random
Zhang et al [26]	Esophageal neoplasms	0.94 (0.92-0.96)	0.85 (0.73-0.92)	6.40 (3.38-12.11)	0.06 (0.04-0.10)	98.88 (39.45-247.87)	0.97 (0.95-0.98)	Random
Xie et al [25]	Gastric cancer	0.89 (0.85-0.93)	0.93 (0.88-0.97)	13.4 (7.3-25.5)	0.11 (0.07-0.17)	NR	0.94 (0.91-0.98)	Random
Xie et al [25]	Invasion depth of gastric cancer	0.82 (0.78-0.85)	0.90 (0.82-0.95)	8.4 (4.2-16.8)	0.20 (0.16-0.26)	NR	0.90 (0.87-0.93)	Random
Chen et al [19]	Gastric cancer	0.86 (0.75-0.92)	0.90 (0.84-0.93)	NR	NR	NR	0.94^f^	NR

a PLR: positive likelihood ratio.

b NLR: negative likelihood ratio.

c DOR: diagnostic odds ratio.

d AUC: area under the curve.

e NR: not reported.

f 95% CIs were not reported.

### Grading of Evidence

We evaluated the reliability of each outcome through GRADE. Results showed that the quality was evaluated as very low for 44.1% of the outcomes and low for 55.9% of the outcomes. Our research found that the sensitivity and specificity of Barrett neoplasia, esophageal squamous cell carcinoma, *Helicobacter pylori* infection, chronological gastritis, colorectal polyp, gastrointestinal ulcer, and gastrointestinal hemorrhage had low credibility. The other outcomes had very low credibility. Generally speaking, the primary defects were indirectness and imprecision. These problems were caused by the different AI models and training methods used in the original literature, and there were also differences in the selection of recognition samples. Endoscopists in different regions used different samples and chose different AI algorithms to train and test the models, making the synthesized results less credible. Detail can be seen in Table S4 in Multimedia Appendix 1.

## Discussion

### Principal Findings

In this study, we conducted a systematic review of the current use of AI in endoscopic diagnosis, assessing the quality of research and meta-analyses conducted in this field. AI has been studied and applied in upper gastrointestinal endoscopy, colorectal endoscopy, capsule endoscopy, and laryngoscopy. The meta-analysis results showed that AI has high sensitivity and specificity for these types of endoscopy. However, the overall evidence level of the outcomes was low.

In previous studies, AI could effectively assist in sedation and training in the operation process of upper digestive tract examination [39,40]. The earliest research we examined was conducted in 2007, when computers were trained to identify esophageal cancer [41]. At that time, the research only distinguished malignant and nonmalignant esophageal tissues in vitro.

With the rise of AI and the continuous upgrading of training methods, the application of AI in gastrointestinal endoscopy, including esophageal cancer, gastric cancer, and *Helicobacter pylori* infection, has been widely studied. In addition to the ordinary white light examination, computer-aided systems have shown a certain diagnostic value in stained and magnifying endoscopic imaging [42,43]. Moreover, some studies have found that trained models have research value in diagnosing gastric cancer’s infiltration depth [44].

A study in 2022 compared the diagnostic value of computer-aided systems and professional endoscopists in gastric cancer images through retrospective data and found no significant difference in the diagnostic rate between the two groups [45]. This shows that AI aid is not inferior to endoscopists in image diagnosis. Wu conducted a single-center randomized controlled trial and found that the missed diagnosis rate of gastric adenoma could be significantly reduced using AI [46]. Multi-center randomized controlled studies are still needed for further analysis in the future.

AI has been widely studied in colorectal endoscopy. A meta-analysis showed that AI could effectively improve adenoma detection rate [7]. However, another meta-analysis based on real-world research reached the opposite conclusion [47]. The findings of our study proposed that AI has a noticeable effect in identifying intestinal lesions. However, many problems still need to be effectively addressed, particularly in terms of clinical implementation and practical translation.

In November 2023, the team at West China Hospital led a 12-center study with more than 10,000 patients [48]. This randomized controlled trial compared the relationship between AI-assisted and routine examinations in the missed diagnosis rate of esophageal lesions. The results showed that AI could not significantly improve the missed diagnosis rate of esophageal lesions. Many teams are constantly developing, improving and trying to use AI models in clinics. As mentioned above, although AI has been shown to have a significant effect in many studies, there has been an increase in research regarding the failure of AI to significantly improve the effectiveness of endoscopy in the clinical situation. In the application process, we found that the recognition threshold of AI greatly affected its application value. We are explored the possiblity of classifying patients according to some baseline information or endoscopic mucosal background images and then continuously optimized the AI recognition threshold according to the risk stratification of different patients. This approach aimed to achieve individualized endoscopic examinations and improve overall identification accuracy [49-51].

Moreover, the economic impact of a large-scale rollout of AI systems in clinical work on patients and health care institutions must be further studied. In addition, the differences in validation sets make it difficult to truly achieve accurate side-by-side comparisons when evaluating the capabilities of different AI models, which may lead to biased results. We believe it would be beneficial to produce an open platform that includes test data sets from different parts and different lesions of the gastrointestinal tract so that researchers can test the effectiveness of AI recognition in the future.

This study has several strengths. According to our preliminary understanding, the umbrella evaluation of using AI in endoscopic applications must be revised. To a certain extent, we have filled this blank. Second, we conducted a strict analysis and discussion following the PRISMA guidelines. Third, two researchers conducted all analyses, and the results were reliable.

There are also some limitations to this study. First, various computer-aid models have certain heterogeneity, and this could not be avoided in the analysis. Therefore, our results are a general summary of the current technology. Second, we could not gather the data of some unpublished studies. Third, the limited number of studies made it difficult to do further subgroup analyses. Fourth, we only included studies reported in English, which might have introduced some biases to our study.

### Conclusions

This study found that AI has high diagnostic value in endoscopy. These findings provide a theoretical basis for the development and evaluation of AI-assisted systems, aimed at assisting endoscopists in conducting examinations, thereby improving patient health outcomes. However, it is worth noting that there is no convincing high-quality evidence in the existing research and further research is needed in the future.

## Supplementary material

10.2196/56361 Multimedia Appendix 1 Additional statistics.

10.2196/56361 Checklist 1 PRISMA (Preferred Reporting Items for Systematic reviews and Meta-Analyses) checklist.

## Abbreviations

AI: artificial intelligence
AMSTAR: A Measurement Tool to Assess Systematic Reviews
EUS: endoscopic ultrasound
GRADE: Grading of Recommendations, Assessment, Development, and Evaluation
PRISMA: Preferred Reporting Items for Systematic reviews and Meta-Analyses
PROBAST: Prediction Model Risk of Bias Assessment Tool
QUADAS: Quality Assessment of Diagnostic Accuracy Studies
