# Supplementary material for: Diagnostic Accuracy of Artificial Intelligence in Endoscopy: Umbrella Review
Source: JMIR Med Inform. 2024 Jul 15;12:e56361. doi: 10.2196/56361 (PMC11296324; doi:10.2196/56361)
Supplement: Multimedia Appendix 1 [file medinform-v12-e56361-s001.docx]

**Supplementary Material**

Table S1: Search term

| **#** | **Searches** | **Results** |
| --- | --- | --- |
| **PubMed** | | |
| 1 | "Systematic Review" [Publication Type] | 244308 |
| 2 | "Meta-Analysis" [Publication Type] | 189527 |
| 3 | #1 OR #2 | 105577 |
| 4 | "Artificial Intelligence" [MeSH Terms] OR "Artificial Intelligence" [Title/Abstract] | 202125 |
| 5 | "Machine Learning" [MeSH Terms] OR "Machine Learning" [Title/Abstract] | 126469 |
| 6 | "Deep Learning" [MeSH Terms]OR "Deep Learning" [Title/Abstract] | 56662 |
| 7 | "Neural Network" [MeSH Terms]OR "Neural Network" [Title/Abstract] | 71579 |
| 8 | "Computer"[MeSH Terms]OR " Computer" [Title/Abstract] | 261350 |
| 9 | #4 OR #5 OR #6 OR #7 OR #8 | 536688 |
| 10 | "Endoscopy" [MeSH Terms]OR " Endoscopy" [Title/Abstract] | 2388456 |
| 11 | "Colonoscopy" [MeSH Terms]OR " Colonoscopy" [Title/Abstract] | 51330 |
| 12 | "Upper gastrointestinal endoscopy" [MeSH Terms]OR "Upper gastrointestinal endoscopy" [Title/Abstract] | 5083 |
| 13 | "Endoscopic retrograde cholangiopancreatography" [MeSH Terms]OR "Endoscopic retrograde cholangiopancreatography" [Title/Abstract] | 10886 |
| 14 | "laryngoscopy" [MeSH Terms]OR "laryngoscopy" [Title/Abstract] | 18889 |
| 15 | #10 OR #11 OR #12 OR #13 OR #14 | 469422 |
| 16 | #3 AND #9 AND #15 | 133 |
| **Embase** | | |
| 1 | ' systematic review '/exp | 440033 |
| 2 | ' meta analysis '/exp | 296328 |
| 3 | #1 OR #2 | 567495 |
| 4 | ' Artificial Intelligence '/exp OR ' Artificial Intelligence ':ti,ab,kw | 98426 |
| 5 | ' Machine Learning'/exp OR ' Machine Learning':ti,ab,kw | 445070 |
| 6 | ' Deep Learning '/exp OR ' Deep Learning':ti,ab,kw | 68318 |
| 7 | ' Neural Network '/exp OR ' Neural Network ':ti,ab,kw | 84462 |
| 8 | ' Computer '/exp OR ' Computer ':ti,ab,kw | 435538 |
| 9 | #4 OR #5 OR #6 OR #7 OR #8 | 905159 |
| 10 | ' Endoscopy '/exp OR Endoscopy :ti,ab,kw | 832953 |
| 11 | ' Colonoscopy '/exp OR  Colonoscopy:ti,ab,kw | 113003 |
| 12 | ' Upper gastrointestinal endoscopy '/exp OR  Upper gastrointestinal endoscopy:ti,ab,kw | 27529 |
| 13 | ' Endoscopic retrograde cholangiopancreatography '/exp OR  Endoscopic retrograde cholangiopancreatography:ti,ab,kw | 18529 |
| 14 | ' laryngoscopy '/exp OR ' laryngoscopy ':ti,ab,kw | 32613 |
| 15 | #10 OR#11 OR #12 OR #13 OR #14 | 498 |
| 16 | #3 AND #9 AND #15 | 6932 |

| **WOS** | | |
| --- | --- | --- |
| 1 | (TS= (meta analysis)) OR (TS=(system review)) | 1950067 |
| 2 | (((((TS= Artificial intelligence)) OR TS= (Machine Learning)) OR TS=( Deep Learning)) OR TS=(Neural Network)) OR TS=( Computer) | 8787028 |
| 3 | ((((TS=( Endoscopy)) OR TS=( Colonoscopy )) OR TS=( Upper gastrointestinal endoscopy )) OR TS=( Endoscopic retrograde cholangiopancreatography )) OR TS=( laryngoscopy ) | 260690 |
| 4 | #1 AND #2 AND #3 AND #4 | 2249 |
| 5 |  |  |

| **K** | | |
| --- | --- | --- |
| 1 | ("Artificial intelligence"):ti,ab,kw | 6 |
| 2 | (" Machine Learning "):ti,ab,kw | 9325917 |
| 3 | (" Deep Learning "):ti,ab,kw | 143435 |
| 4 | ("Neural Network "):ti,ab,kw | 574518 |
| 5 | (" Computer "):ti,ab,kw | 725 |
| 6 | #1 OR #2 OR #3 OR #4 OR #5 | 350 |

Table S2: Methodological characteristics of included study

| Study | Aim | Outcome | Tools for assessing the risk of the bias | Registered Number |
| --- | --- | --- | --- | --- |
| Tan | Detection of Barrett’s esophagus | Sn, Sp, AUC, Acc | QUADAS-2 | NR |
| Ma | Detection of esophagus cancer | Sn, Sp, AUC, Acc | QUADAS‑2 | N |
| Bang | Detection of Helicobacter Pylori Infection | Sn, Sp, AUC, Acc | QUADAS-2 | CRD42020175957 |
| Shi | Detection of Chronic atrophic gastritis | Sn, Sp, AUC, Acc | QUADAS-2 | CRD42022371134 |
| Guidozzi | Detection of Barrett’s esophagus and cancer | Sn, Sp, AUC, Acc | NR | N |
| Jahagirdar | Detection of Ulcerative colitis | Sn, Sp, AUC, Acc | NR | N |
| Keshtkar | Detection of Colorectal polyp and Cancer | Sn, Sp, AUC, Acc | PROBAST | 10.17605/OSF.I O/QJ7EU |
| Bang | Detection of ulcers, polyps, celiac disease, bleeding, and hookworm | Sn, Sp, AUC, Acc | QUADAS-2 | CRD42021253454 |
| Soffer | Detection of ulcers, polyps, celiac disease, bleeding, and hookworm | Sn, Sp, AUC, Acc | QUADAS-2 | N |
| Gomes | Detection of gastrointestinal stromal tumor | Sn, Sp | QUADAS-2 | CRD42023418987. |
| Zurek | Detection of lesions in the larynx. | Sn, Sp, AUC, Acc | QUADAS-2 | CRD42021282843 |
| Bai | Prediction of invasion depth of colorectal cancer or neoplasms | Sn, Sp, AUC, PLR, NLR, DOR | QUADAS-2 | CRD42022331046 |
| Qin | Detection of erosion/ulcer, gastrointestinal bleeding and polyps/cancer | Sn, Sp, AUC, Acc | QUADAS-2 | N |
| Mohan | Detection of GI ulcers | Sn, Sp, AUC, Acc | NR | N |
| Bang | Detection of diminutive colorectal polyps | Sn, Sp, AUC, Acc | QUADAS-2 | CRD42021232189 |
| Lui | Detection of Colorectal polyp and Cancer | Sn, Sp, AUC, Acc | QUADAS-2 | [CRD42020167274](https://www.crd.york.ac.uk/prospero/display_record.php?ID=CRD42020167274) |
| Lui | Detection of Gastric and esophageal neoplastic lesions and Helicobacter pylori | Sn, Sp, AUC, Acc | QUADAS-2 | N |
| Visaggi | Detection of Barrett's neoplasia | Sn, Sp, AUC, Acc | QUADAS | N |
| Zhang | Detection of Esophageal cancer and neoplasm | Sn, Sp, AUC, Acc | QUADAS-2 | N |
| Xie | Detection of Gastric cancer and prediction invasion depth | Sn, Sp, AUC, Acc | QUADAS-2 | CRD42021227312 |
| Chen | Detection of Early Gastric Cancer | Sn, Sp, AUC, Acc | QUADAS-2 | CRD42020193223 |

Table S3: Detailed evaluation of studies with AMSTAR 2

| Study | 1 | 2 | 3 | 4 | 5 | 6 | 7 | 8 | 9 | 10 | 11 | 12 | 13 | 14 | 15 | 16 | Final rating |
| --- | --- | --- | --- | --- | --- | --- | --- | --- | --- | --- | --- | --- | --- | --- | --- | --- | --- |
| Tan | Y | pY | N | pY | Y | Y | Y | Y | Y | N | Y | Y | Y | Y | Y | Y | Low |
| Ma | Y | pY | N | Y | Y | N | Y | Y | Y | N | Y | Y | Y | Y | Y | Y | Low |
| Bang | Y | Y | N | Y | Y | N | Y | pY | Y | N | pY | Y | Y | Y | Y | Y | Moderate |
| Shi | Y | Y | N | Y | Y | Y | Y | Y | Y | N | pY | Y | Y | Y | Y | Y | Moderate |
| Guidozzi | Y | pY | N | Y | N | N | pY | pY | N | N | pY | Y | Y | N | N | Y | Critical low |
| Jahagirdar | Y | pY | N | Y | Y | Y | Y | pY | N | N | pY | N | Y | Y | Y | Y | Critical low |
| Keshtkar | Y | Y | Y | Y | Y | Y | Y | pY | N | N | pY | Y | Y | Y | N | Y | Critical low |
| Bang | Y | Y | N | Y | Y | Y | Y | pY | Y | N | pY | Y | Y | Y | Y | Y | Moderate |
| Soffer | Y | pY | N | Y | Y | N | pY | Y | Y | N | pY | N | Y | Y | N | Y | Critical low |
| Gomes | Y | Y | N | pY | Y | N | Y | pY | Y | N | pY | N | Y | Y | N | Y | Critical low |
| Zurek | Y | Y | Y | Y | Y | Y | Y | pY | Y | N | pY | N | N | N | Y | Y | Critical low |
| Bai | Y | Y | Y | Y | Y | Y | Y | Y | Y | N | Y | Y | Y | Y | Y | Y | Moderate |
| Qin | Y | pY | N | Y | Y | N | N | Y | Y | N | pY | Y | Y | N | N | Y | Critical low |
| Mohan | Y | N | N | Y | N | Y | Y | pY | N | N | pY | Y | N | N | Y | Y | Critical low |
| Bang | Y | Y | N | Y | Y | Y | Y | Y | Y | N | pY | N | Y | N | Y | Y | Moderate |
| Lui | Y | Y | N | pY | Y | N | Y | Y | Y | N | pY | N | Y | N | N | Y | Critical low |
| Lui | Y | Y | N | pY | Y | N | pY | pY | Y | N | pY | N | N | Y | Y | Y | Critical low |
| Visaggi | Y | pY | Y | Y | Y | Y | Y | pY | Y | N | Y | Y | Y | Y | Y | Y | Moderate |
| Zhang | Y | pY | N | pY | N | Y | Y | Y | Y | N | Y | Y | N | Y | Y | Y | Critical low |
| Xie | Y | Y | N | pY | Y | Y | Y | Y | Y | N | Y | Y | Y | Y | Y | Y | Moderate |
| Chen | Y | Y | N | pY | Y | Y | Y | Y | Y | N | Y | Y | Y | Y | Y | Y | Moderate |

1: Did the research questions and inclusion criteria for the review include the components of PICO? 2: Did the report of the review contain an explicit statement that the review methods were established prior to the conduct of the review and did the report justify any significant deviations from the protocol? 3: Did the review authors explain their selection of the study designs for inclusion in the review? 4: Did the review authors use a comprehensive literature search strategy? 5: Did the review authors perform study selection in duplicate? 6: Did the review authors perform data extraction in duplicate? 7: Did the review authors provide a list of excluded studies and justify the exclusions? 8: Did the review authors describe the included studies in adequate detail? 9: Did the review authors use a satisfactory technique for assessing the risk of bias (RoB) in individual studies that were included in the review? 10: Did the review authors report on the sources of funding for the studies included in the review? 11: If meta-analysis was performed, did the review authors use appropriate methods for statistical combination of results? 12: If meta-analysis was performed, did the review authors assess the potential impact of RoB in individual studies on the results of the meta-analysis or other evidence synthesis? 13: Did the review authors account for RoB in primary studies when interpreting/discussing the results of the review? 14: Did the review authors provide a satisfactory explanation for, and discussion of, any heterogeneity observed in the results of the review? 15: If they performed quantitative synthesis did the review authors carry out an adequate investigation of publication bias (small study bias) and discuss its likely impact on the results of the review? 16: Did the review authors report any potential sources of conflict of interest, including any funding they received for conducting the review?

Table S4: Detailed credibility evaluation of outcomes with GRADE

| Disease | Outcomes | Downgrade | | | | | GRADE |
| --- | --- | --- | --- | --- | --- | --- | --- |
|  |  | Risk of Bias | lnconsistency | Indirectness | Imprecision | Publication bias |  |
| Barrett's neoplasia | SE | No serious | Serious | Serious | No serious | No serious | Low |
| Esophageal squamous cell carcinoma | SE | No serious | Serious | Serious | No serious | No serious | Low |
| Abnormal intrapapillary capillary loops | SE | No serious | Serious | Serious | No serious | Serious | Very Low |
| Gastroesophageal reflux disease | SE | No serious | Serious | Serious | No serious | Serious | Very Low |
| Gastric cancer | SE | Serious | Serious | Serious | No Serious | No serious | Very Low |
| Invasion depth of gastric cancer | SE | No serious | No Serious | Serious | No serious | Serious | Very Low |
| Helicobacter Pylori Infection | SE | No serious | Serious | Serious | No Serious | No serious | Low |
| Chronic atrophic gastritis | SE | No serious | Serious | Serious | No Serious | No serious | Low |
| Ulcerative colitis | SE | Serious | Serious | Serious | No Serious | Serious | Very Low |
| Colorectal polyp | SE | No Serious | Serious | Serious | No Serious | No Serious | Low |
| Colorectal cancer | SE | Serious | Serious | Serious | No Serious | No Serious | Very Low |
| Invasion depth of early CRC | SE | Serious | Serious | Serious | No Serious | No Serious | Very Low |
| Gastrointestinal Ulcer | SE | No serious | Serious | Serious | No Serious | No serious | Low |
| Gastrointestinal Hemorrhage | SE | No serious | Serious | Serious | No Serious | No serious | Low |
| Gastrointestinal stromal tumors | SE | No serious | Serious | Serious | No Serious | Serious | Very Low |
| Healthy laryngeal tissue | SE | No serious | Serious | Serious | No Serious | Serious | Very Low |
| Benign and Malignant Lesions | SE | No serious | Serious | Serious | No Serious | Serious | Very Low |
| Barrett's neoplasia | SP | No serious | Serious | Serious | No serious | No serious | Low |
| Esophageal squamous cell carcinoma | SP | No serious | Serious | Serious | No serious | No serious | Low |
| Abnormal intrapapillary capillary loops | SP | No serious | Serious | Serious | No serious | Serious | Very Low |
| Gastroesophageal reflux disease | SP | No serious | Serious | Serious | No serious | Serious | Very Low |
| Gastric cancer | SP | Serious | Serious | Serious | No Serious | No serious | Very Low |
| Invasion depth of gastric cancer | SP | No serious | No Serious | Serious | No serious | Serious | Very Low |
| Helicobacter Pylori Infection | SP | No serious | Serious | Serious | No Serious | No serious | Low |
| Chronic atrophic gastritis | SP | No serious | Serious | Serious | No Serious | No serious | Low |
| Ulcerative colitis | SP | Serious | Serious | Serious | No Serious | Serious | Very Low |
| Colorectal polyp | SP | No Serious | Serious | Serious | No Serious | No Serious | Low |
| Colorectal cancer | SP | Serious | Serious | Serious | No Serious | No Serious | Low |
| Invasion depth of early CRC | SP | Serious | Serious | Serious | No Serious | No Serious | Low |
| Gastrointestinal Ulcer | SP | No serious | Serious | Serious | No Serious | No serious | Low |
| Gastrointestinal Hemorrhage | SP | No serious | Serious | Serious | No Serious | No serious | Low |
| Gastrointestinal stromal tumors | SP | No serious | Serious | Serious | No Serious | Serious | Low |
| Healthy laryngeal tissue | SP | No serious | Serious | Serious | No Serious | Serious | Low |
| Benign and Malignant Lesions | SP | No serious | Serious | Serious | No Serious | Serious | Low |
| SE: sensitity; SP: specificity   1. Risk of Bias: We assigned ‘serious’ when studies with a low QUADAS2(QUADAS) score comprised a large proportion. In order to ensure the reliability of the results, if the study does not report QUADAS2(QUADAS) score, we will set the results as serious. 2. lnconsistency: We assigned ‘lnconsistency’ by accounting heterogeneity measured by the I^2^ statistic (>50%), variability in point estimates, and extent of overlap in confidence intervals. In order to ensure the reliability of the results, if the study does not report heterogeneity, we will set the results as serious. 3. Indirectness: We assigned ‘indirectness’ when the outcome was derived from study populations that differed from those of interest or key information of methods such as detection method or sample source are different. 4. Imprecision: We assigned ‘imprecision’ when the sample size was too small (<1000 cases) or the confidential interval (CI) was too large. 5. Publication bias: We assigned ‘detected’ when substantial asymmetry was observed in the funnel plot, or when the p value was <0.05 in Deek’s test or Egger’s test. If the inspection is not carried out, we also regard it as risky. In order to ensure the reliability of the results, if the study does not report biased results, we will set the results as serious. | | | | | | | |
